# Broad infectivity of *Leidynema appendiculatum* (Nematoda: Oxyurida: Thelastomatidae) parasite of the smokybrown cockroach *Periplaneta fuliginosa* (Blattodea: Blattidae)

**DOI:** 10.1002/ece3.3948

**Published:** 2018-03-23

**Authors:** Sota Ozawa, Koichi Hasegawa

**Affiliations:** ^1^ Department of Environmental Biology College of Bioscience & Biotechnology Chubu University Kasugai Japan

**Keywords:** cockroach, host range, invasive species, oxyurida, parasitic nematode

## Abstract

Host specificity of parasites is important for the understanding of evolutionary strategies of parasitism that would be a basis of predictions of the disease expansion when parasitized hosts invade new environments. The nematode order Oxyurida is an interesting parasite group for studying the evolution of parasitism as it includes parasites of both invertebrates and vertebrates. In our survey, we found that the smokybrown cockroach *Periplaneta fuliginosa* was primarily infected with only one nematode species *Leidynema appendiculatum*. In two cases, *L. appendiculatum* was isolated from two additional cockroach species *Pycnoscelus surinamensis,* sold in Japan as a reptile food, and *Blatta lateralis,* captured in the field and cultured in the laboratory. Inoculation of *L. appendiculatum* into three additional cockroach species *P. japonica*,* Blattella nipponica*, and *P. surinamensis* also resulted in parasitism. Infection prevalence was high, and timing of postembryonic development from hatched nematode larva to mature adult in these hosts was identical with that in *P. fuliginosa*. While ecological interactions strongly determine the host range, such broad infectivity is still possible in this parasitic nematode.

## 1 INTRODUCTION

The host specificity of parasites is important as it reflects the evolutionary strategy of parasitism. The nematode order Oxyurida consists of a number of families that are divided into two superfamilies. The first superfamily Oxyuroidea, which consists of vertebrate parasitic nematodes (also called pinworms that include the human parasite *Enterobius vermicularis*), and second Thelastomatoidea, which consists of invertebrate parasitic nematodes (Carreno, 2014; Nadler et al., 2007). Nematodes belonging to the order Oxyurida share a simple infection cycle: Nematode eggs laid by adult females are deposited within their host feces and released by defecation, next ingestion of eggs by new host individuals leads to infection (Adamson, 1994; Ozawa et al., 2016). While Oxyurida parasites sometimes cause diseases in humans, such as colitis, perianal abscess, and ectopic infections in females (Ariyarathenam et al., 2010; St Georgiev, 2005), these conditions are not regarded as serious compared to infectious diseases caused by another gastrointestinal worms including Onchocerciasis, Elephantiasis, and Ancylostomiasis (Bungiro & Cappello, 2004; Pilotte, Unnasch, & Williams, 2017). Moreover, Oxyurida parasites in invertebrates are seemingly harmless to their hosts (Adamson, 1994; Ozawa et al., 2014), but consequences of carriage are largely unknown. Cospeciation is well conserved in vertebrates and pinworms (Falk & Perkins, 2013; Hugo, 1999; Murata, Hasegawa, Nakano, Noda, & Yanai, 2002). However, a wide variety of nematode species have been reported from diverse invertebrate hosts and that do not seemingly reflect host/parasite cospeciation events (Adamson & van Waerebeke, 1992; Jex, Schneider, & Cribb, 2006a; Jex, Schneider, Rose, & Cribb, 2007). The evolutionary strategies of parasitism might differ between Oxyuroidea and Thelastomatoidea.

The family Thelastomatidae have been reported for more than forty Blattodea species (Adamson & van Waerebeke, 1992; Ozawa et al., 2014, 2016; Sriwati, Ozawa, Morffe, & Hasegawa, 2016). We reported earlier for the first time that the smokybrown cockroach *Periplaneta fuliginosa* in Japan was infected with only one nematode species *Leidynema appendiculatum* with high prevalence (Ozawa et al., 2014). However, *L. appendiculatum* is reported as a cosmopolitan nematode species, which has been isolated from many Blattaria hosts known as sanitary pests, including *P. americana* (American cockroach), *P. australasiae* (Australian cockroach), and *Blatta orientalis* (Oriental cockroach) (Adamson & Noble, 1993; Blanco, Lax, Dueñas, Gardenal, & Doucet, 2012; Connor & Adamson, 1998; Shah, 2007). As genotypic characterizations have not been conducted, it is still doubtful whether these nematodes were *L. appendiculatum* or cryptic species.

Here, we show the broad infectivity of *L. appendiculatum* through natural and artificial infection of five cockroach species within three families and two suborders. *L. appendiculatum* was basically isolated from *P. fuliginosa* collected in three area of Japan with high prevalence. Infection prevalence, intensity, and infrapopulation of *L. appendiculatum* in five host cockroach species were similar. Moreover, the developmental timing of this nematode from hatched larvae to matured adult was identical. While ecological interactions with *P. fuliginosa* seemingly strongly determine the host range of *L. appendiculatum*, such broad infectivity is still possible in this parasitic nematode.

## 2 MATERIALS AND METHODS

### 2.1 Cockroach strains and rearing

The following laboratory‐culturing cockroach strains (seven strains from five species in three genus, two subfamily) used in this experiment were reared as described by Ozawa et al., 2014, (1) *Periplaneta fuliginosa* EE, (2) *P. fuliginosa* UF, (3) *P. japonica* Miyoshi, (4) *Pycnoscelus surinamensis* Pet, (5) *P. surinamensis* Yaedake, (6) *Blattella nipponica* CU, (7) *Blatta lateralis* KX.

We used two independent strains of *P. fuliginosa*:* P. fuliginosa* EE strain was supplied by the Earth Environmental Service (Ako, Hyogo Prefecture, Japan) and reared since 2012. The *P. fuliginosa* UF strain, also laboratory‐culturing strain, has been established and reared for more than 60 years in the Philip G. Koehler laboratory, University of Florida. *P. japonica*, the Japanese cockroach or Yamato cockroach, is a native of Japan and belonging to the same *Periplaneta* genus with *P. fuliginosa*. The *P. japonica* Miyoshi strain was established from 10 adult males and 10 females collected at the Miyoshi city (Aichi Prefecture, Japan) in June 2012. The *P. surinamensis* Pet strain, sold for pet reptile food, was obtained by Japanese online shop (no detailed information obtained). The *P. surinamensis* Yaedake strain was established from three adult females hand‐picked at the Yaedake (Okinawa prefecture, Japan) in March 2013. *B. nipponica* is also a native of Japan. The *B. nipponica* CU was collected at the Chubu University campus (Kasugai, Japan) during April to September 2014 and 2017; because this species was impossible to be reared in laboratory for over a year, all individuals were collected and reared only temporary before experiments. The *B. lateralis* KX strain was established from three adult females collected at Knoxville (Tennessee, USA) in July 2013.

Nematode‐free cockroach strains were established as follows: oothecae oviposited were collected, surface wiped with 70% ETOH, and kept in a plastic dish until hatching. Hatched nymphs were transferred into the plastic cage and reared. *P. japonica* Miyoshi, *B. nipponica* CU, and *P. surinamensis* Yaedake were originally nematode‐free. Individuals of the established nematode‐free strains were often dissected to confirm the absence of parasitic nematodes.

### 2.2 Cockroach dissection and parasitic nematode observation

The hindgut of cockroach adult male and female was extracted, placed in a Syracuse watch glass containing cockroach Ringer's solution (NaCl 9.32 g, KCl 0.77 g, NaHCO_3_ 0.18 g, NaH_2_PO_4_ 0.01 g, CaCl_2_ 0.5 g in 1 L of distilled water), and split longitudinally with tweezers to release nematodes. Nematode species, sex, and stage were observed under a stereomicroscope (SMZ600, Nikon, Japan). When detailed morphological information was needed, nematodes were picked up with a mouth pipette, transferred onto an agar pad (Shaham, 2006), covered, and sealed with a silicon grease‐rimmed coverslip for viewing by Nomarski DIC optics (Eclipse E600, Nikon).

Because we found that four cockroach strains (*P. fuliginosa* EE, *P. fuliginosa* UF, *P. surinamensis* Pet, and *B. lateralis* KX) were originally infected with *L. appendiculatum*, we analyzed the infrapopulation of nematode male, female, and juvenile by the software Quantitative Parasitology 3.0 (Rózsa, Reiczigel, & Majoros, 2000).

### 2.3 Molecular identification and phylogenetic analysis

Genomic DNA from a single female nematode was extracted using Qiagen DNeasy Blood & Tissue Kit (Qiagen, USA) following manufacturer's instructions. The D2/D3 expansion segment of 28S ribosomal RNA gene (D2/D3 LSU) and partial fragment of small subunit of ribosomal RNA gene (SSU) were amplified using the universal primers D2A (5′‐ACA AGT ACC GTG AGG GAA AGT TG‐3′) and D3B (5′‐TCG GAA GGA ACC AGC TAC TA‐3′) (Nunn, 1992), and nSSU_F_07 (5′‐AAA GAT TAA GCC ATG CAT G‐3′) and nSSU_R_26 (5′‐CAT TCT TGG CAA ATG CTT TCG‐3′) (Blaxter et al., 1998, The Blaxter Lab website: http://www.nematodes.org/research/barcoding/sourhope/nemoprimers.shtml). The amplified DNA fragment was purified from agarose gels with NucleoSpin^®^ Gel and PCR Clean‐up (MACHEREY‐NAGEL, Germany). Samples were submitted to Hokkaido System Science Co., Sapporo, Japan, for sequencing from both strands, using the same PCR primers. Sequences were deposited in GenBank NCBI (http://www.ncbi.nlm.nih.gov/genbank/).

For the phylogenetic analysis, several sequences from Thelastomatidae were selected: *L. appendiculatum* JQ343844, EU365630, and KC540759. *L. portentosae* GQ401114 and EF180073 were selected as outgroup. ClustalW multiple alignment was conducted in BioEdit version 7.2.6 (Hall, 1999), and sequence alignments were trimmed automatically by trimAI with default setting (Capella‐Gutiérrez, Silla‐Martínez, & Gabaldón, 2009). Phylogenetic trees were constructed from evolutionary distances using the maximum likelihood (ML) method using the Mega 6.0 software (Tamura, Stecher, Peterson, Filipski, & Kumar, 2013). Based model was D2/D3 LSU: Hasegawa‐Kishino‐Yano model (Hasegawa, Kishino, & Yano, 1985); SSU: Jukes‐Cantor model (Jukes & Cantor, 1969). Phylogenetic robustness was inferred by bootstrap analysis using 1,000 iterations (Felsenstein, 1985).

Pairwise comparisons of % differences (*D*) between each sequence combination were performed using the formula *D* = (*M*/*L*) × 100 (Chilton, Gasser, & Beveridge, 1995), where *M* is the number of alignment positions at which the two sequences have a base in common, and *L* is the total number of alignment positions.

### 2.4 Artificial infection test of parasitic nematodes

Artificial infection experiments were performed in four nematode‐free cockroach strains, *P. fuliginosa* EE, *P. japonica* Miyoshi, *P. surinamensis* Yaedake, *B. nipponica* CU. *L. appendiculatum* adult females were collected from nematode‐infected *P. fuliginosa* EE as described above. The transferred *L. appendiculatum* gravid females were placed in a Syracuse watch glass containing cockroach Ringer's solution and cut in the center by sterilized scalpel blade to release eggs (~150 eggs per individual). Released eggs were kept in the cockroach Ringer's solution for 14 days at 25°C until all reached to the L2 resting stage. The last instar of nematode‐free cockroaches was fasted for 3 days before artificial infection. Ten fasted cockroaches were reared in a cylindrical plastic case (130 mm diameter × 225 mm height) with 0.4 g of the bait mixed with about 500 of the L2 resting stage nematode eggs. After artificial infection, cockroaches were fed with bait without nematode eggs *ad libitum*. To observe the nematode prevalence (% of the infected cockroaches among all cockroaches examined), intensity (mean number of nematodes in the infected cockroaches, excluding the number “zero” of uninfected host), and developmental stages, one or two cockroaches of each group from *P. fuliginosa* EE were dissected 1, 3, 7, 14, 21, 28, and 35 days after infection, or one or two cockroaches of each group from *P. japonica*,* B. nipponica*, and *P. surinamensis* were dissected every week (from 7 days until 35 days after infection). All nematodes isolated in these experiments were mounted on the agar pad (described above) and observed by Nomarski DIC optics (Eclipse E600, Nikon).

After experimental infection, the rest of *P. japonica* was pooled and reared for several generations, and then checked the infrapopulation of *L. appendiculatum* male, female, and juvenile by the Quantitative Parasitology 3.0 (Rózsa et al., 2000).

## 3 RESULTS

### 3.1 Two exceptional cases of *L. appendiculatum* hosts

We dissected 14 species of cockroaches collected in the field, and as well laboratory‐cultured strains. Basically *L. appendiculatum* were isolated only from *P. fuliginosa* in our experiments. We always isolated only *L. appendiculatum* from laboratory‐cultured (Ozawa et al., 2014) and field‐captured (Table S1) *P. fuliginosa* with high prevalence. In this study, we showed that the two different laboratory strains of *P. fuliginosa* EE (Japan) and UF (USA) were infected with *L. appendiculatum* with 100% prevalence (Table 1). As these two *P. fuliginosa* strains were established independently and maintained for a long period in different countries and laboratories, we conclude that this parasitic association of *L. appendiculatum* with *P. fuliginosa* is quite stable.

**Table 1:** Infrapopulation of *Leidynema appendiculatum* in five cockroach strains

Host cockroach			*L. appendiculatum* infection data				
Strain and sex^a^	*N*	Body size^b^	Stage	Prevalence (Cl)^c^	Mean intensity (Cl)^d^	Median intensity (CI)^e^	V/M ratio^f^
*P.f*. EE Male	10	25.7 ± 1.4	♂	50.0 (22.3–77.8)	1.00 (uncertain)	1.0 (1–1)	0.56
			♀	70.0 (38.1–91.3)	2.43 (1.43–4.57)	2.0 (1–7)	2.62
			J	100 (70.9–100)	7.60 (3.40–15.30)	4.0 (1–20)	11.73
*P.f*. EE Female	15	26.6 ± 1.8	♂	86.7 (60.3–97.6)	1.15 (1.00–1.46)	1.0 (1–1)	0.43
			♀	100 (77.8–100)	9.20 (5.6–19.00)	8.0 (3–12)	14.57
			J	93.3 (69.8–99.7)	15.43 (10.71–19.79)	15.0 (8–24)	5.94
*P.f*. UF Male	20	27.2 ± 1.7	♂	70.0 (47.5–86.0)	1.14 (1.00–1.43)	1.0 (1–1)	0.61
			♀	70.0 (47.5–86.0)	2.14 (1.43–2.93)	1.5 (1–3)	1.72
			J	50.0 (29.3–70.7)	2.70 (1.90–3.80)	2.0 (2–5)	2.28
*P.f*. UF Female	22	29.4 ± 1.9	♂	81.8 (61.1–93.5)	1.06 (1.00–1.17)	1.0 (1–1)	0.25
			♀	90.9 (70.9–98.3)	4.45 (3.65–5.25)	4.0 (3–5)	1.24
			J	45.5 (20.8–71.8)	3.60 (2.20–5.10)	3.0 (1–6)	3.06
*P.s*. Pet Female	10	18.5 ± 2.5	♂	90.0 (55.4–99.5)	1.00 (uncertain)	1.0 (1–1)	0.11
			♀	100 (70.9–100)	3.50 (2.70–4.30)	3.0 (2–5)	0.59
			J	100 (70.9–100)	18.90 (12.40–28.80)	13.5 (7–39)	10.05
*B.l*. KX Male	18	21.8 ± 1.4	♂	88.9 (67.0–98.0)	1.00 (uncertain)	1.0 (1–1)	0.12
			♀	94.4 (72.9–99.7)	2.65 (2.06–3.12)	3.0 (2–3)	0.67
			J	55.6 (33.0–76.4)	3.70 (2.30–5.60)	2.0 (2–8)	3.86
*B.l*. KX Female	19	24.3 ± 1.5	♂	78.9 (55.4–92.5)	1.00 (uncertain)	1.0 (1–1)	0.22
			♀	100 (82.5–100)	4.47 (3.37–5.74)	4.0 (3–5)	1.75
			J	94.7 (67.2–99.9)	11.39 (7.83–16.22)	9.0 (3–13)	8.24
*P.j*. AI Male	22	24.9 ± 2.1	♂	45.5 (26.1–66.2)	1.00 (uncertain)	1.0 (1–1)	0.57
			♀	59.1 (38.3–77.8)	2.23 (1.62–2.92)	2.0 (1–3)	1.62
			J	72.7 (50.0–87.4)	4.19 (2.69–5.94)	3.5 (1–5)	3.86
*P.j*. AI Female	20	24.8 ± 2.9	♂	40.0 (20.1–62.8)	1.00 (uncertain)	1.0 (1–1)	0.63
			♀	65.0 (42.4–83.3)	2.15 (1.46–2.85)	1.0 (1–4)	1.68
			J	75.0 (52.6–89.6)	8.27 (5.27–11.47)	6.0 (3–14)	7.14

a *P.f*. EE, *P. fuliginosa* EE strain. *P.f*. UF, *P. fuliginosa* UF strain. *P.s*. Pet, *P. surinamensis* Pet strain. *B.l*. KX, *B. lateralis* KX strain. *P.j*. AI, *P. japonica* artificial infection strain. All individuals examined in this experiment were adult and 100% nematode infection prevalence except *P.j*. AI female (91%).

b Host cockroach body size, average ± *SD* (mm).

c Sterner's exact method, confidence limits for the population prevalence (95% Confidence limits).

d Bootstrap (BCa) method, confidence limits for the mean intensity (95% Confidence limits), except for adult male nematode from *P.f*. EE Male (93.8% confidence level).

e Exact confidence limits for the median intensity (95% Confidence limits).

f Variance to mean ratio is calculated including uninfected hosts as well.

Interestingly, we also found two rare cases in the cockroach species *P. surinamensis* and *B. lateralis*. We established three strains of *P. surinamensis*; the first strain *P. surinamensis* Pailand was infected with unknown nematode species *Suifunema* sp. with almost 100% of prevalence (data not shown). The second strain, *P. surinamensis* Yaedake, was free from nematode, but the third *P. surinamensis* Pet was infected with *L. appendiculatum* with 100% prevalence. We established one strain of *B. lateralis* KX and found that was also infected with *L. appendiculatum* with 100% prevalence. We confirmed that these nematodes were *L. appendiculatum* from morphological characteristics of the adult male and female (data not shown).

### 3.2 Molecular identification and phylogenetic analysis of *L. appendiculatum*

**Figure 1:**
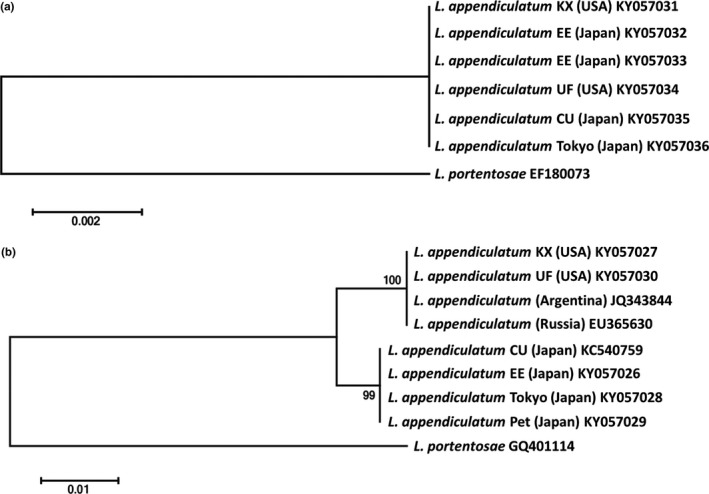
Maximum likelihood (ML) trees inferred from (a) 18S SSU rDNA and (b) D2D3 LSU rDNA for *Leidynema appendiculatum* isolated from cockroach hosts. *Leidynema portentosae* was used as outgroup. Values at the nodes correspond to bootstrap probability

We obtained ribosomal DNA sequence data, D2/D3 LSU and SSU from *L. appendiculatum* isolated from cockroach strains used in these experiments including *P. fuliginosa* CU (Ozawa et al., 2014), *P. fuliginosa* EE, *P. fuliginosa* UF, *P. surinamensis* Pet, and *B. lateralis* KX. We also obtained sequence data for *L. appendiculatum* Tokyo 20140825SB, isolated from *P. fuliginosa* captured in Minato city, Tokyo in 2014 (Table S1). These data were deposited in the NCBI GenBank (accession numbers, Table 2). Several published sequences of *Leidynema* spp. were included in this phylogenetic analysis. All SSU sequences of *L. appendiculatum* were 100% identical (Figure 1a), whereas D2/D3 LSU sequences showed unique polymorphisms that divided into two clades. One clade contained *L. appendiculatum* isolated from cockroaches in Japan. The other clade included cockroaches from the United States, Russia, and Argentina (Figure 1b). The hosts of Japanese *L. appendiculatum* are *P. fuliginosa* EE, *P. fuliginosa* CU, *P. fuliginosa* Tokyo 140825SB, and *P. surinamensis* Pet. The host species in other countries are *P. fuliginosa* UF (USA), *B. lateralis* KX (USA), *P. americana* (Russia), and *P. americana* (Argentina) (Table 2). Pairwise sequence differences between the two clades were 1.5%, although 0.0% within the clades (Table 3).

**Table 2:** *Leidynema* strains information used in phylogenetic analysis

Nematode	Host cockroach	Original Country	D2/D3 Accession No.	SSU Accession No.	References
*L. appendiculatum* EE	*Periplaneta fuliginosa* EE	Japan	KY057026	KY057032	This study
*L. appendiculatum* UF	*P. fuliginosa* UF	USA	KY057030	KY057034	This study
*L. appendiculatum* Pet	*Pycnoscelus surinamensis* pet	Japan	KY057029	KY057033	This study
*L. appendiculatum* KX	*Blatta lateralis* KX	USA	KY057027	KY057031	This study
*L. appendiculatum* Tokyo	*P. fuliginosa* Tokyo	Japan	KY057028	KY057036	This study
*L. appendiculatum* CU	*P. fuliginosa* CU	Japan	KC540759	KY057035	Ozawa et al. (2014)
*L. appendiculatum*	*P. americana*	Argentina	JQ343844	–	Blanco et al. (2012)
*L. appendiculatum*	*P. americana*	Russia	EU365630	–	Spiridonov, direct submission
*L. portentosae*	*Gromphadorhina portentosa*	–	GQ401114	EF180073	Nadler et al. (2007)
					Spiridonov et al. (2009)

**Table 3:** Pairwise differences (%) in the D2/D3 sequence between nine samples of the genus *Leidynema* (Leidy, 1850) Chitwood, 1932

	1	2	3	4	5	6	7	8	9
1. *L. appendiculatum* CU (Japan) KC540759	–								
2. *L. appendiculatum* EE (Japan) KY057026	0.0	–							
3. *L. appendiculatum* Pet (Japan) KY057029	0.0	0.0	–						
4. *L. appendiculatum* Tokyo (Japan) KY057028	0.0	0.0	0.0	–					
5. *L. appendiculatum* UF (USA) KY057030	1.5	1.5	1.5	1.5	–				
6. *L. appendiculatum* KX (USA) KY057027	1.5	1.5	1.5	1.5	0.0	–			
7. *L. appendiculatum* (Russia) EU365630	1.5	1.5	1.5	1.5	0.0	0.0	–		
8. *L. appendiculatum* (Argentina) JQ343844	1.5	1.5	1.5	1.5	0.0	0.0	0.0	–	
9*. L. portentosae* GQ401114	9.1	9.1	9.1	9.1	9.4	9.4	9.4	9	–

### 3.3 Infrapopulation of *L. appendiculatum* in different cockroach strains

We analyzed the prevalence, intensity, and infrapopulation structure of *L. appendiculatum* males, females, and juveniles in four cockroach strains: *P. fuliginosa* EE, *P. fuliginosa* UF, *P. surinamensis* Pet, and *B. lateralis* KX. All cockroaches in the laboratory‐cultured strains were infected with *L. appendiculatum* with 100% prevalence. We also confirmed the infrapopulation structures were similar in each cockroach strain. First, the distributions of female and juvenile nematodes are positive skewed (Mean > Median); second, the number of nematode males was basically one or zero; third, the mean and median intensity of nematodes in female hosts is higher than that in male hosts; and fourth, there was no difference in nematode prevalence between male and female cockroach hosts (Table 1).

### 3.4 Artificial infection of *L. appendiculatum* in *P. fuliginosa*

**Figure 2:**
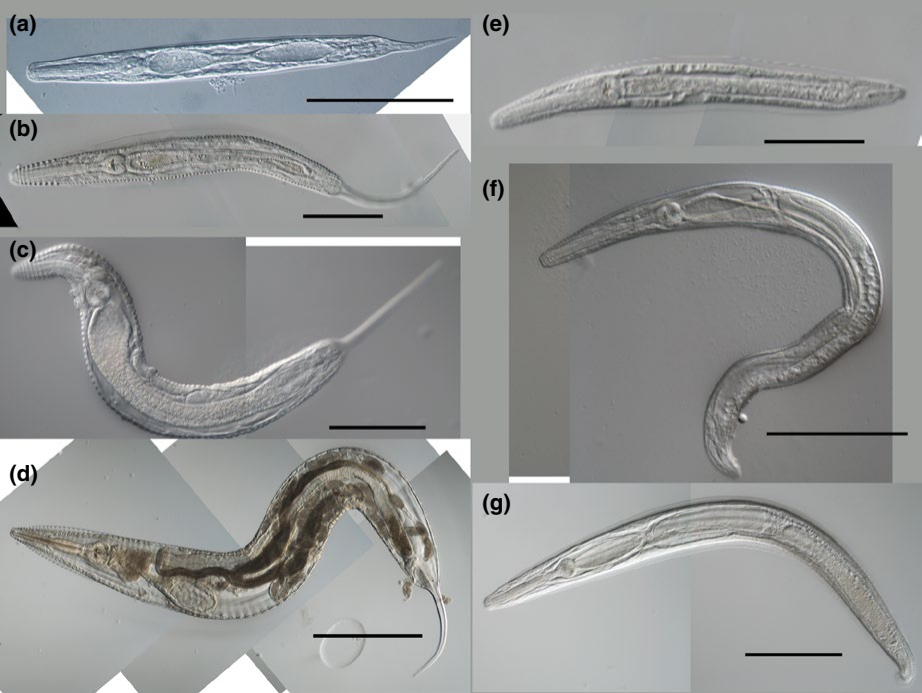
Developmental stage of *L. appendiculatum*. (a) Larva, 1 day after infection. (b) Female larva, after 14 days of infection. (c) Female larva, after 21 days of infection. (d) Matured adult female, after 28 days of infection. (e) Male larva, after 14 days of infection. (f) Male larva, after 21 days of infection. (g) Matured adult male, after 28 days of infection. Scale bars, (a, e) 100 μm, (b, c, f, g) 200 μm, (d) 500 μm

Next, we inoculated *L. appendiculatum* into *P. fuliginosa* to monitor nematode prevalence and developmental timing from the L2 stage egg to mature adult. Just 1 day after infection, several nematode larvae appeared in the cockroach hindgut (Figure 2a). After 3 days of infection, differences of male/female morphology were not evident at these larval stages. Seven days postinfection, few nematodes started molting. After molting, morphological differences between male and female appeared. The spicule and copulatory apparatus appeared in males’ round‐shaped tails that resembles tails of adult males (Figure 2e). Female tail shape became long and filiform (Figure 2b). After 14 days of infection, nematodes started molting (Figure S1A–D) and sexual dimorphism became prominent despite lack of sexually maturity (Figure 2c,f). At this timing, 16 nematodes were molting and showing female character, and four were male character. The rest of the nematodes (*N* = 31) were still larvae and without sexual dimorphisms. At 21 days postinfection, reproduction systems of the nematodes were maturing or matured (Figure S1E–H). At 28 days after infection, males and females had reached adulthood, and eggs were visible in adult female uteri (Figure 2d,g). At 35 days after infection, a small number of larvae appeared in the cockroach hindgut that seemed to be a new infection with a second‐generation nematodes.

**Figure 3:**
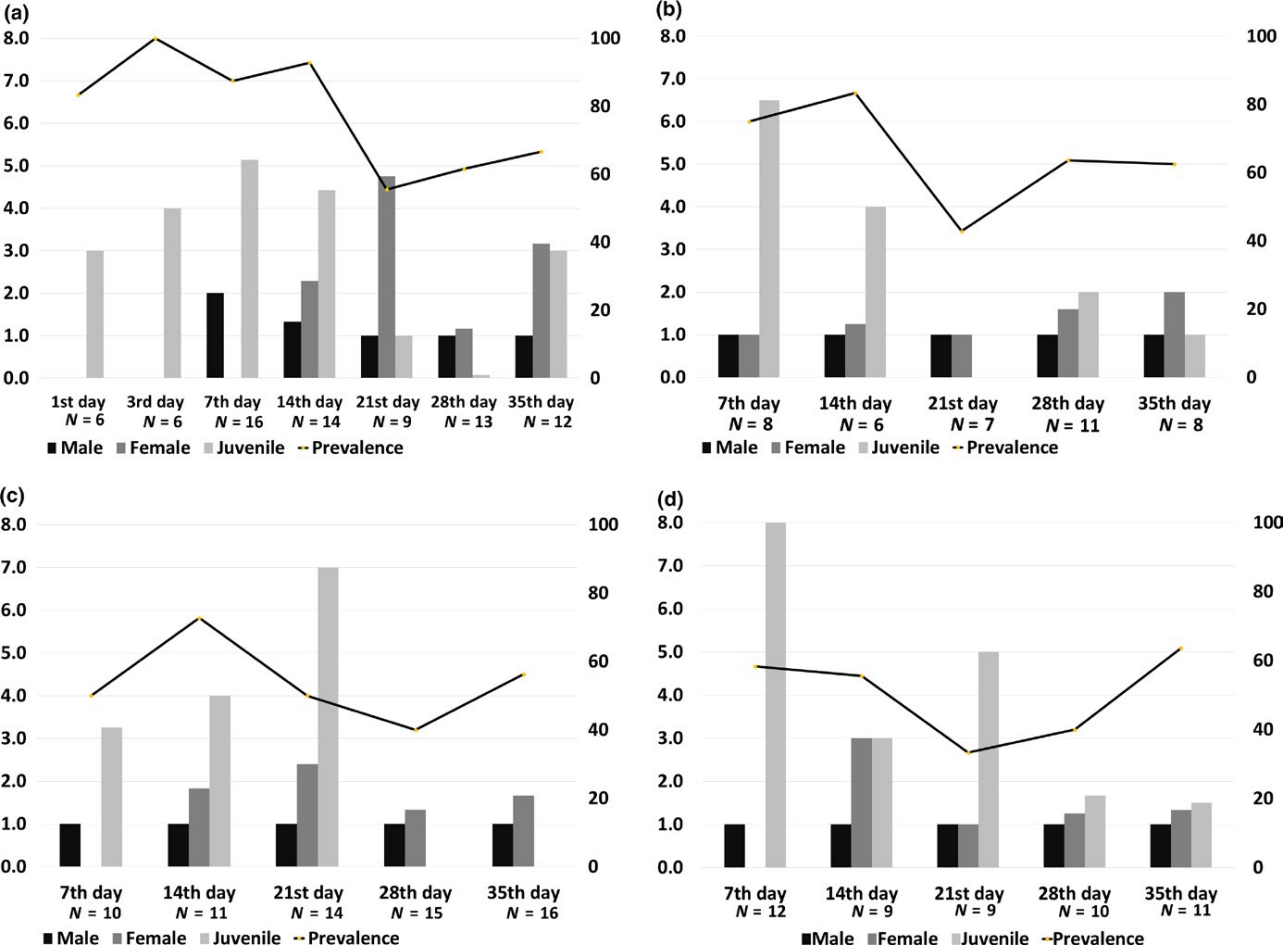
Nematode intensity (%) (bars, left *y*‐axis) and infection prevalence (lines, right *y*‐axis) in each cockroach host, (a) *P. fuliginosa*, (b) *P. japonica*, (c) *B. nipponica*, and (d) *P. surinamensis*

Figure 3a shows the infection prevalence of *L. appendiculatum* and mean intensity (mean number of nematodes in the infected cockroaches, excluding the number “zero” of uninfected host) of male, female, and juvenile nematodes in a cockroach hindgut at 1 to 35 days after infection. We counted male/female when nematodes appeared sexually dimorphic (at or after the 7th day of infection, Figure 2b,e) and classified as juvenile when the dimorphism was not clear (before 7th day of infection, Figure 2a). As indicated in Figure 2, several hatched vermiform larvae appeared immediately from the 1st day, and the first sexual dimorphism appeared in males on the 7th day. The mean intensity of males was two on the 7th day, but mostly did not exceed one after 21 days of infection. The average nematode prevalence over the course of the experiment (from 1st day to 35th day) was 78% (*N* = 76).

### 3.5 Artificial infection of *L. appendiculatum* in other cockroaches

Next, we inoculated *L. appendiculatum* into the cockroach species, *P. japonica*,* B. nipponica,* and *P. surinamensis*, which belong respectively to three families (Blattidae, Blattellidae, Blaberidae) and two suborders (Blattoidea, Blaberoidea). We observed the nematode number, developmental stage, and sex. Figure 3b–d shows the infection prevalence of *L. appendiculatum* (%) and mean intensity (uninfected “zero” data were excluded) of male, female, and juvenile nematodes in a cockroach hindgut at 7 to 35 days after infection.

Although average nematode prevalence over the course of the study was lower in *P. japonica* (65%, *N* = 40), *B. nipponica* (53%, *N* = 66), and *P. surinamensis* (51%, *N* = 51) when compared with that in *P. fuliginosa* (78%, *N* = 76), *L. appendiculatum* successfully infected all three hosts. The developmental timings were the same as that observed in *P. fuliginosa* (Figure 2b–d). The mean intensity of male, female, and juvenile nematodes in cockroach hindgut at each timing was similar, although no juvenile nematodes were observed in *B. nipponica* at 28 and 35 days postinfection (Figure 3c).

After these artificial infection experiments, the remaining *P. japonica* were combined and reared for several generations under the same conditions as our laboratory‐cultured strains (see Materials and Methods) and then checked for infection prevalence. The population of *L. appendiculatum* in adult male and female cockroaches was assessed. Infection prevalence was 91% (*N* = 22) in adult males and 90% (*N* = 20) in adult females, and infection intensity was similar to the other cockroaches in cultured in the laboratory (Table 1).

## 4 DISCUSSION

We have dissected 14 cockroach species including *P. fuliginosa* (Smokybrown cockroach), *P. japonica* (Yamato cockroach), *P. americana* (American cockroach), *P. australasiae* (Australian cockroach), *Blattella germanica* (German cockroach), *B. nipponica* (Japanese forest roach), *B. lituricollis*,* Blatta lateralis*,* Pycnoscelus surinamensis* (Surinam cockroach), *P. indicus* (Indicus cockroach), *Onychostylus pallidiolus*,* Lobopterella dimidiatipes*,* Opisthoplatia orientalis*,* Panesthia angustipennis spadica* (data not shown). These species were collected in the field as well as cultured in the laboratory. Although we have not finished identification and description of all parasitic nematodes yet, host specificity of the nematodes belonging to the family Thelastomatidae was seemingly high. Species combinations of cockroach host and parasitic nematode were almost fixed except for two exceptions described in this work. We always isolated *L. appendiculatum* from *P. fuliginosa* with high prevalence. *L. appendiculatum* was not isolated from the other cockroaches with the two exceptions; one of our three *P. surinamensis* strains and one of our one *B. lateralis* strain (Table 1). Captured *B. lateralis* were concluded to be originally infected with *L. appendiculatum* rather than to be contaminated during rearing in laboratory. More investigations are needed to understand the patterns of infection of the parasitic nematodes in *B. lateralis* in the field. Our finding that *B. lateralis* can act as a host of parasitic nematodes might be a case showing the broad host range of *L. appendiculatum*.

In the case of *B. lateralis*, we only checked one strain for these experiments. However, we did check more than ten individuals of wild *P. surinamensis* as well as laboratory‐cultured strains. All parasitic nematodes isolated from *P. surinamensis* were unknown species *Suifunema* sp. (data not shown) except for one laboratory‐cultured strain *P. surinamensis* Pet (Table 1). As this cockroach reproduces parthenogenetically, grows, and reproduces quickly, it is easy to maintain without any special condition. Therefore in Japan, *P. surinamensis* is popular for pet reptile breeders as a food of their animals. As *P. fuliginosa* is the most widely distributed cockroach in Japan, there are likely many chances for contamination with the parasitic nematode from *P. fuliginosa* if the cockroaches are cultured in environments of general households or pet shops.

**Figure 4:**
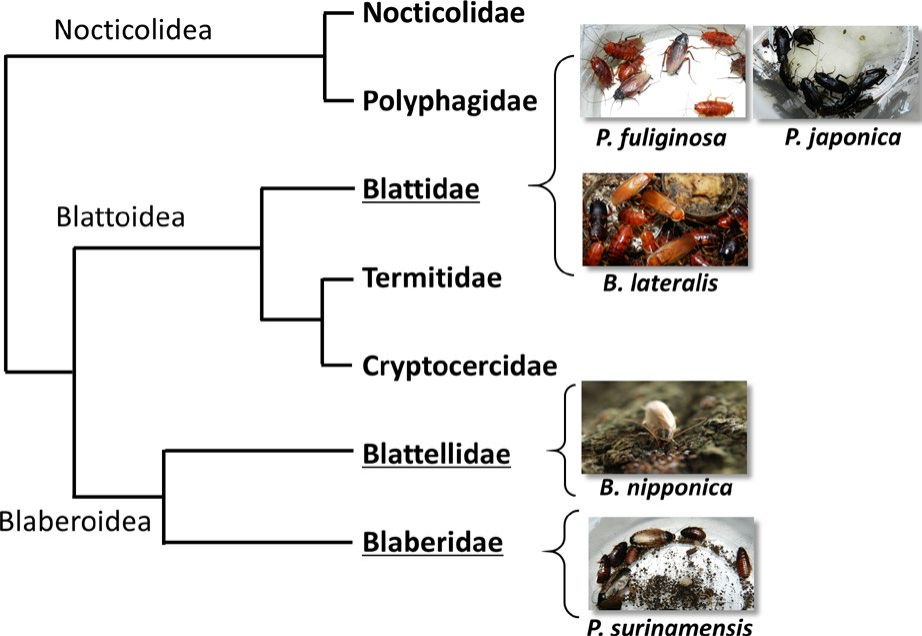
Phylogenetic relation of five cockroach species examined in these experiments, constructed based on Inward, Beccaloni, & Eggleton (2007). These species belong to three families (Blattidae, Blattellidae, Blaberidae) and two suborders (Blattoidea, Blaberoidea). Blattodea was believed to separate with Mantodea from common ancestor between 250 and 200 mya. Two suborders, Blattoidea and Blaberoidea, were thought to separate between 200 and 150 mya (Misof et al. 2014). American cockroach *P. americana* another major sanitary pest and reported as a host of *L. appendiculatum* also belongs to the family Blattidae (Grimaldi & Engel, 2005)

We present here that *L. appendiculatum* was capable of infecting three cockroach species, *P. japonica*,* B. nipponica*, and *P. surinamensis*. The infectivity of *L. appendiculatum* is quite broad and includes five cockroach species, three families in two suborders (Figure 4). *P. japonica* is a Japanese domestic species and lives mainly in northern area moving between indoors to outdoors (Tanaka & Tanaka, 1997; Tanaka & Uemura, 1996). *P. surinamensis* is estimated to have originated in the Indo‐Malayan region and is now distributed worldwide in tropical and subtropical regions (Kramer & Brenner, 2009). We found *P. japonica* and *P. surinamensis* were infected with specific nematode *Protrellus* sp. and *Suifunema* sp., respectively, with high prevalence (data not shown). *B. nipponica* is a Japanese domestic cockroach and morphologically resembles *B. germanica*, the world sanitary pest, however, with differences in ecological traits; *B. nipponica* mainly lives in the outdoors under fallen leaves in forests and grassland of southwestern Japan (Asahina, 1991; Tsuji, 1985). Interestingly, *B. nipponica* is always free from parasitic nematodes.

We also confirmed that *L. appendiculatum*, when inoculated artificially into *B. nipponica* could persist in the host hindgut and lay eggs but could not be re‐infected. This is likely because *B. nipponica* does not eat feces. An infection cycle for parasitic nematodes might not be established. This result matches with our data that *B. nipponica* captured in the field is not infected with parasitic nematodes at all. Therefore, we conclude that *L. appendiculatum* is not a native parasite of *B. nipponica*. It was surprising that *L. appendiculatum* can infect a cockroach species that has a different ecological and systematic position than *P. fuliginosa*.

Although infection prevalence of *L. appendiculatum* within these three cockroaches was less than that in *P. fuliginosa*, values exceeded 50% and the developmental timing of nematode maturation resembled that seen in *P. fuliginosa* (Figure 3). We believe that there was no difference in infection opportunities as all cockroaches consumed all of the given bait within a few days. These results might suggest that *L. appendiculatum* has evolved as a parasite of *P. fuliginosa*, but that its infection mechanism makes it capable of infecting other cockroach species accidentally when exposed.

The remarkable features of *L. appendiculatum* we could reveal in these experiments are (1) it has a broad infectivity and (2) it has a strong association with a cockroach species that is currently spreading and inhabiting worldwide. *P. fuliginosa* inhabits all prefectures of Japan, although mainly central and western Japan (Nakano, 2013; The Japanese Society of Pestology, 2015; Tsuji, 1975). As the cockroaches examined in this experiment originally live in different habitats, there would be few opportunities to replace their original parasite in nature. The field survey revealed host sharing of thelastomatid nematodes and suggested that host specificity is dictated largely not by their taxonomy but by the host ecology (Jex et al., 2006a,b, 2007). As the habitat of thelastomatid nematodes is absolutely limited to the host cockroach intestine, parasite and host have cospeciated over a long historical period to form a tight association. On the other hand, this nematode group has a simple parasitic ecology; eggs are discharged together with the host feces and infect new host individuals directly by oral infection without intermediate hosts. Because of such simple mechanisms of nematode infections, interspecific host exchange might occur more frequently in nature than we thought and that might have promoted its broad infectivity. As we confirmed that nematode‐free *P. japonica* was infected with *L. appendiculatum* when cultured together with *L. appendiculatum*‐infected *P. fuliginosa* (data not shown), nematode exchange between species could be established by feeding feces of other cockroach species. Recently, surveys of parasitic nematodes in native and invasive cockroaches in Galápagos Islands were reported. *L. appendiculatum* was isolated from invasive species *P. americana*,* P. australasiae*, and *P. surinamensis* with relatively low prevalence (14%, 31%, and 6%, respectively; Sinnott, Carreno, & Herrera, 2015). *Cephalobellus ovumglutinosus*, first described from worldwide distributed species *B. germanica* (van Waerebeke, 1978), was isolated from endemic and invasive cockroaches (Sinnott et al., 2015). As there is no record of *P. fuliginosa* in these islands, these cockroaches can be also considered nematode carriers contributing to their spreading.

Adamson proposed that ancestors of oxyurid nematodes were diversifying in invertebrates long before the parasites of vertebrates, and that they probably arose in diplopods or blattarians (Adamson, 1989, 1994). Cospeciation of host and parasite was well conserved in primates and pinworm nematodes (Hugo, 1999). Host specificity influences the expansion of parasitic diseases when the host invades a new environment. Thus, understanding host range might contribute valuable information (Agosta, Janz, & Brooks, 2010). For example, the human pinworm *Enterobius vermicularis* only causes itching in the anal area of its native human host, but can be fatal in chimpanzees *Pan troglodytes* (Murata et al., 2002). In addition, parasitic nematodes greatly affect the composition of microbial communities in cockroaches. It is obvious that there are direct and indirect interactions between hosts and parasites (Vicente, Ozawa, & Hasegawa, 2016). This nematode is also a good model system for understanding the mechanisms and evolution of mutualistic relationships between parasites and hosts.

## CONFLICT OF INTEREST

None Declared.

## AUTHOR CONTRIBUTION

KH and SO planned experiments; SO and KH performed experiments; KH and SO analyzed data; KH contributed reagents and material; KH and SO wrote the study. All authors read and approved the final manuscript.
