# Supplementary figures and images for: Broad infectivity of Leidynema appendiculatum (Nematoda: Oxyurida: Thelastomatidae) parasite of the smokybrown cockroach Periplaneta fuliginosa (Blattodea: Blattidae)
Source: Ecol Evol. 2018 Mar 23;8(8):3908–18. doi: 10.1002/ece3.3948 (PMC5916268; doi:10.1002/ece3.3948)

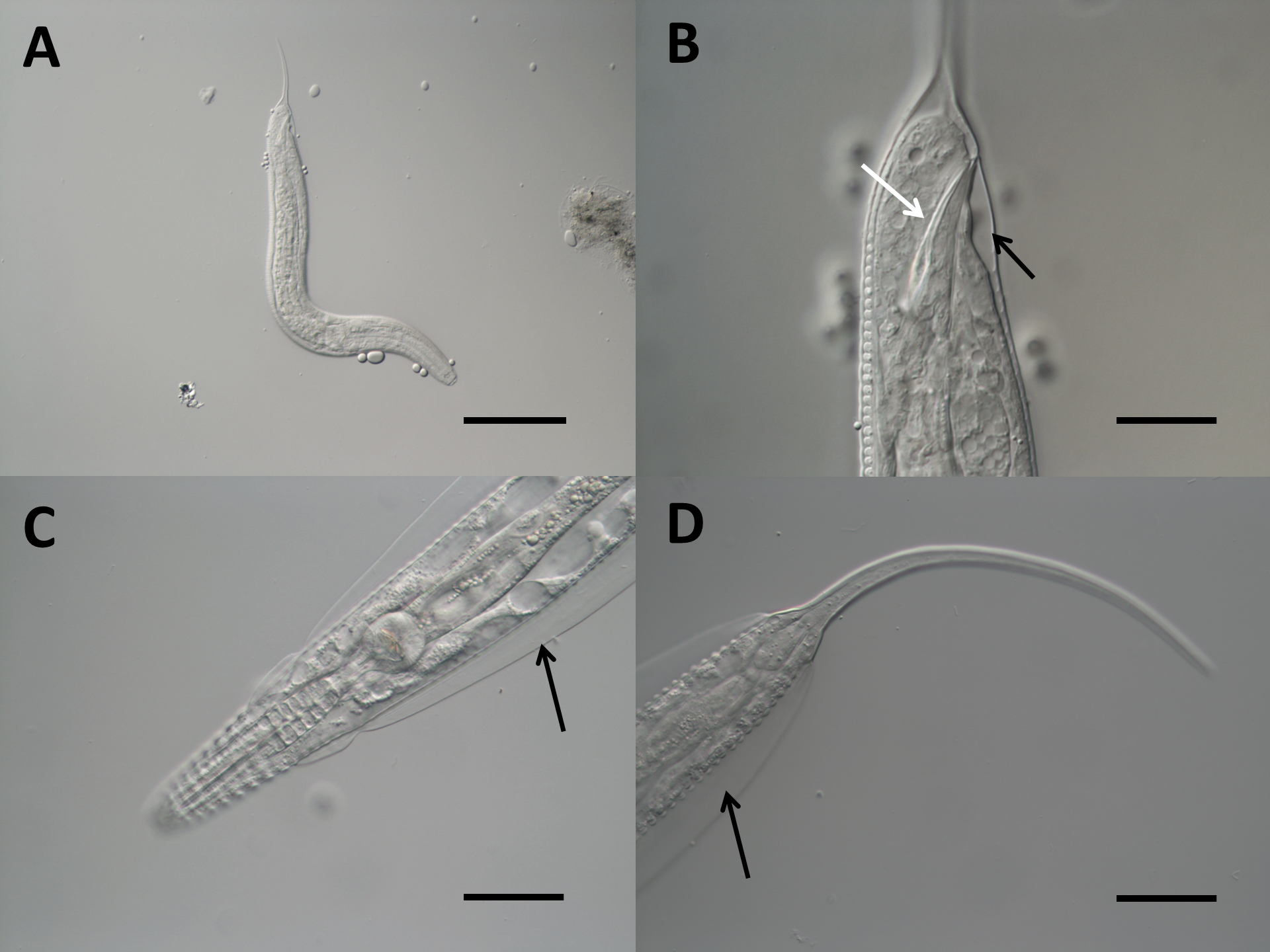

Supplement: Supplementary file 1 [file ECE3-8-3908-s001.tif]

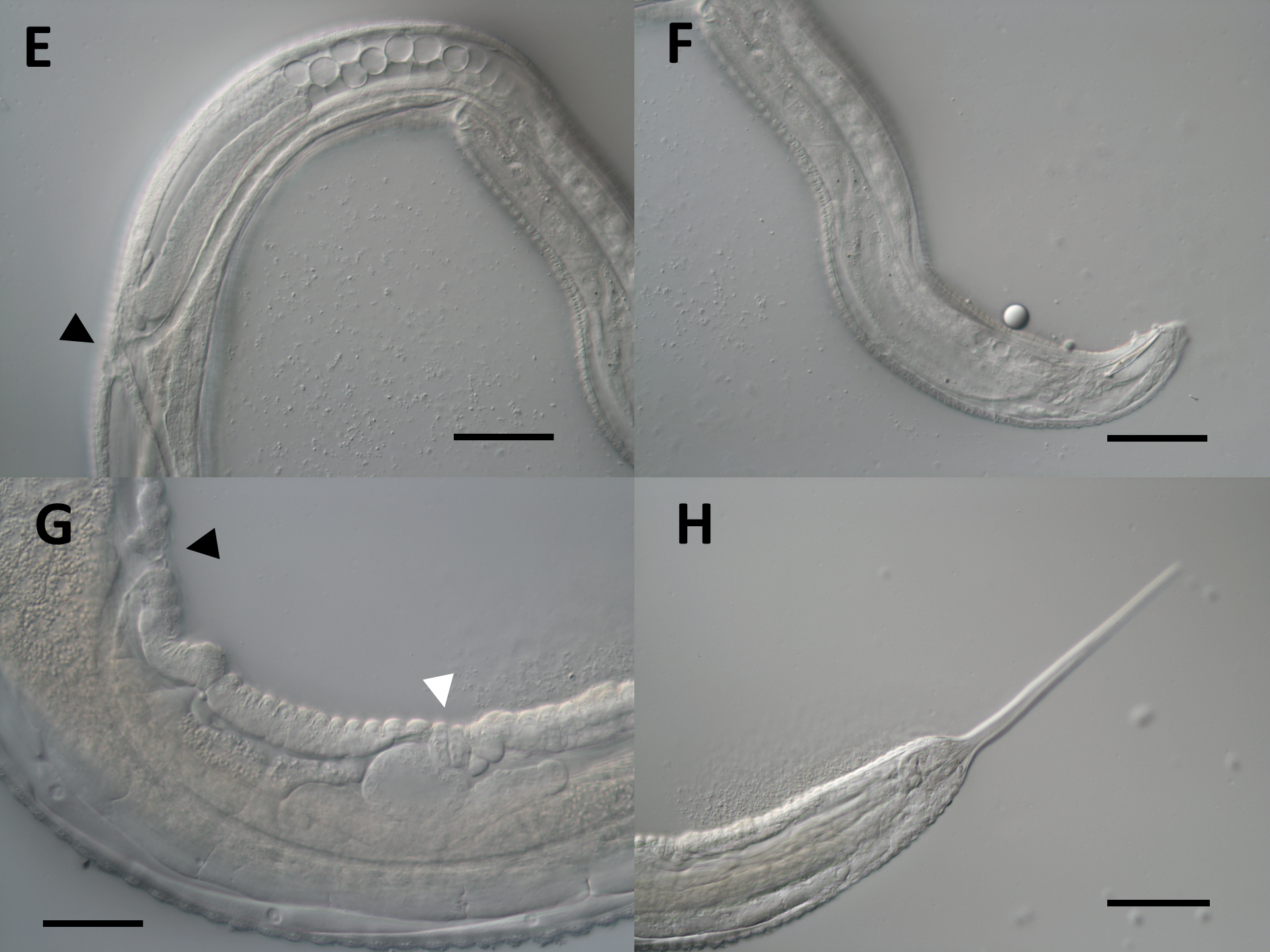

Supplement: Supplementary file 2 [file ECE3-8-3908-s002.tif]
