# Supplementary material for: Broad infectivity of Leidynema appendiculatum (Nematoda: Oxyurida: Thelastomatidae) parasite of the smokybrown cockroach Periplaneta fuliginosa (Blattodea: Blattidae)
Source: Ecol Evol. 2018 Mar 23;8(8):3908–18. doi: 10.1002/ece3.3948 (PMC5916268; doi:10.1002/ece3.3948)
Supplement: Supplementary file 3 [file ECE3-8-3908-s003.docx]

**Table S1** Infrapopulation of *L. appendiculatum* in *P. fuliginosa* collected in the field

Host cockroach *Leidynema appendiculatum* infection data

Stage and Sex^1^ N Body size^2^ Male^3^ Female^3^  Juvenile^3^ Prevalence (%)^4^

Tokyo Total 29 15.8 ± 8.5 1 2.5 3.5 55.2

Adult Male 4 28.5 ± 1.0 1 1.7 4.0 100

Adult Female 2 30.5 ± 2.1 1 7.0 8.0 50

Nymph 23 12.3 ± 5.5 1 2.6 1.0 48

Chubu Total 37 19.4 ± 7.0 1 3.8 5.1 32.4

Adult Male 2 24.0 ± 0.0 1 4.0 5.5 100

Adult Female 6 27.7 ± 0.8 1 3.5 5.7 67

Nymph 29 17.3 ± 6.4 1 0.0 4.3 21

Kyusyu Total 16 25.6 ± 5.9 1 5.4 9.0 81.3

Adult Male 3 30.7 ± 2.1 1 3.0 1.5 100

Adult Female 4 30.8 ± 1.0 1 10.3 15.5 100

Nymph 9 21.7 ± 4.7 1 4.2 5.3 66.7

^1^ Cockroaches captured in Tokyo (N=29), Chubu (N=37), and Kyusyu (N=16) area.

^2^ Host cockroach body size, average ± SD (mm).

^3^ Mean number of nematodes in the infected cockroaches, excluding the number “zero” of uninfected host.

^4^ % of the infected cockroaches among all cockroaches examined.
